# The striatal matrix compartment is expanded in autism spectrum disorder

**DOI:** 10.1186/s11689-025-09596-7

**Published:** 2025-02-15

**Authors:** Jeff L. Waugh, Asim O. A. Hassan, Adrian T. Funk, Joseph A. Maldjian

**Affiliations:** 1 Division of Pediatric Neurology, Department of Pediatrics, University of Texas Southwestern, Dallas, TX USA; 2 Department of Internal Medicine, University of Texas Southwestern, Dallas, TX USA; 3 Department of Radiology, University of Texas Southwestern, Dallas, TX USA; 4 Department of Natural Sciences and Mathematics, University of Texas at Dallas, Dallas, TX USA; 5 Martinos Center for Biomedical Imaging, Massachusetts General Hospital, Charlestown, MA USA

**Keywords:** Striatum, Matrix, Striosome, Autism, Tractography, Connectivity-based Parcellation

## Abstract

**Background:**

Autism spectrum disorder (ASD) is the second-most common neurodevelopmental disorder in childhood. This complex developmental disorder manifests with restricted interests, repetitive behaviors, and difficulties in communication and social awareness. The inherited and acquired causes of ASD impact many and diverse brain regions, challenging efforts to identify a shared neuroanatomical substrate for this range of symptoms. The striatum and its connections are among the most implicated sites of abnormal structure and/or function in ASD. Striatal projection neurons develop in segregated tissue compartments, the matrix and striosome, that are histochemically, pharmacologically, and functionally distinct. Immunohistochemical assessment of ASD and animal models of autism described abnormal matrix:striosome volume ratios, with an possible shift from striosome to matrix volume. Shifting the matrix:striosome ratio could result from expansion in matrix, reduction in striosome, spatial redistribution of the compartments, or a combination of these changes. Each type of ratio-shifting abnormality may predispose to ASD but yield different combinations of ASD features.

**Methods:**

We developed a cohort of 426 children and adults (213 matched ASD-control pairs) and performed connectivity-based parcellation (diffusion tractography) of the striatum. This identified voxels with matrix-like and striosome-like patterns of structural connectivity.

**Results:**

Matrix-like volume was increased in ASD, with no evident change in the volume or organization of the striosome-like compartment. The inter-compartment volume difference (matrix minus striosome) within each individual was 31% larger in ASD. Matrix-like volume was increased in both caudate and putamen, and in somatotopic zones throughout the rostral-caudal extent of the striatum. Subjects with moderate elevations in ADOS (Autism Diagnostic Observation Schedule) scores had increased matrix-like volume, but those with highly elevated ADOS scores had 3.7-fold larger increases in matrix-like volume.

**Conclusions:**

Matrix and striosome are embedded in distinct structural and functional networks, suggesting that compartment-selective injury or maldevelopment may mediate specific and distinct clinical features. Previously, assessing the striatal compartments in humans required *post mortem* tissue. Striatal parcellation provides a means to assess neuropsychiatric diseases for compartment-specific abnormalities. While this ASD cohort had increased matrix-like volume, other mechanisms that shift the matrix:striosome ratio may also increase the chance of developing the diverse social, sensory, and motor phenotypes of ASD.

**Supplementary Information:**

The online version contains supplementary material available at 10.1186/s11689-025-09596-7.

## Introduction

Autism spectrum disorders (ASD) are among the most common neurodevelopmental conditions encountered in medicine [1]. Many types of structural lesions and inherited factors predispose individuals to ASD [2], illustrating that the core clinical features (deficits in language and social function, restricted and rigid interactions and interests, repetitive behaviors, and altered sensory perceptions) [1] are likely a final common pathway of diverse developmental injuries. Prior studies in humans with ASD and in animal models of ASD have demonstrated that specific brain abnormalities covary with features of ASD, including structural abnormalities (cortical thickness [3, 4] and regional volumetry [4–6]) and network abnormalities, such as the power spectrum, global connectivity, and nodal interaction assessments derived from scalp EEG [7, 8] or functional MRI [4, 9]. However, the structural and functional brain abnormalities identified in particular forms of autism (e.g., single gene defects [10] or teratogen exposure [11]) do not generalize to all patients with ASD [2, 12, 13].

To produce the shared phenotypes of autism, the diverse causes of ASD must impinge upon a limited set of anatomic targets [2, 9]. Simultaneously, to produce the range of abnormalities in social, emotional, executive, and motor domains shared in ASD, those shared targets must be functionally generalized. The striatum is one of very few anatomic sites that meet these criteria. The striatum receives projections from nearly every region of the human neocortex [14], and focal striatal injuries can produce discrete deficits in each of the ASD-related domains [15–18]. Deficits in corticostriate projections are common among genetic causes of autism [19, 20], including decreased axon numbers, abnormal excitatory/inhibitory synaptic ratios, and altered long-term synaptic plasticity. Similarly, in the largest neuroimaging analysis of brain volume in ASD (> 3,100 individuals) [6], only four regions had reduced volume in ASD – three were striatal subregions or the primary output nucleus of the striatum. Though each of these lines of investigation implicates the striatum in ASD, and the striatum is neuroanatomically positioned to serve as a convergence point for the many brain abnormalities associated with ASD, understanding how striatal abnormalities might produce the diverse characteristics of autism requires a more precise characterization at the subregional level: the striatal compartments.

The mammalian striatum is divided into distinct but interdigitated compartments known as the matrix and striosome. Distinguishing between the two compartments is impossible with routine histological stains or structural MRI, and they have indistinguishable resting electrophysiologic profiles [21]. However, the two compartments are readily identified using immunohistochemical methods, with more than sixty proteins [22] differentially enriched in one compartment. Matrix and striosome are both comprised of medium spiny neurons (MSNs), but the two populations migrate to the striatum at different times [23], they have relatively-segregated vascular supplies [24], and the striosome is more susceptible to hyperactivation injury following exposure to dopaminergic drugs of abuse [25–27]. The striosome directly inhibits the dopaminergic neurons of the substantia nigra, while the matrix has no similar projection [28]. Matrix MSNs follow the canonical direct–indirect pathway model of dopamine signaling, but striosome MSNs are organized in an inverse pattern [29]. Compartment-selective injury can result from differential physiologic vulnerabilities that are specific to particular developmental stages [30]. Given that matrix and striosome have opposing influences in many behavioral testing paradigms – including reinforcement learning [31], reward and addiction [32], motor action selection [33], and stress-influenced decision making [34] – injuries or developmental derangements that shift the striatal balance toward one compartment may predispose to the motor, limbic, and executive phenotypes common to ASD.

Though limited in number, prior histologic assessments of the striatal compartments in ASD and ASD-like animal models support the hypothesis that the compartments are abnormal in ASD. Kuo and Liu identified indistinct compartment boundaries and an increased matrix:striosome ratio in ASD [35]. Similarly, in a valproate toxicity model of autism [36], the volume of the striosome compartment was reduced by half, the boundaries between the two compartments were less distinct, and MSN populations that are typically segregated between the compartments lost their anatomic specificity. Afferent projections from most areas of cortex [37] are selective for either matrix or striosome. Areas that are highly striosome-selective – basolateral amygdala [38], anterior insula [37, 39], and pregenual anterior cingulate [39] – are also sites that mediate behaviors that may be challenging for individuals with ASD (social engagement [40], empathy and perception of emotions [41], and understanding the intent and motivations of others [42], respectively). We propose that injuries and inherited factors that modify the ratio of matrix:striosome function, either through direct alteration of the striatal compartments or of compartment-specific afferents, may be a neuroanatomic substrate for the behavioral and motor phenotypes of ASD.

Our objectives in this study were to determine i) whether there are compartment-specific differences in volume between ASD and TD, ii) if those differences are present in both caudate and putamen, iii) what parts of the probability distribution include these differences, iv) whether compartment-specific volume differences localize to particular somatotopic zones, and v) if compartment-specific volume differences are attributable to specific bait regions. We investigated these hypotheses in a cohort of 416 individuals (213 matched ASD-control pairs). Using probabilistic diffusion tractography and four decades of anatomic tracing findings in animals, we parcellated the striatum into voxels with matrix-like or striosome-like patterns of structural connectivity. We found a selective expansion in matrix-like voxels in ASD that correlated with the number of autism severity. This expansion occurred throughout the rostral-caudal extent of both caudate and putamen, and was not explained by changes in extra-striate structural connectivity. Factors that increase the absolute or relative function of the striatal matrix may be a cause of some forms of ASD.

## Methods

### Assembling experimental cohorts

We accessed MRI and clinical testing data through the National Institutes of Health Data Archive (NDA) portal. This cohort can be accessed through a study specific identifier (10.15154/z7aa-pz74.). We identified five NDA studies that archived substantial numbers of both ASD and typically developing (TD) control subjects, included robust clinical phenotyping and demographic characterization, and provided diffusion MRI (dMRI) data of similar protocol and resolution: 1, Multimodal developmental neurogenetics of females with ASD (55.6% of all subjects); 2, Atypical late neurodevelopment in autism (23.2%); 3, Biomarkers of developmental trajectories and treatment in ASD (15.0%); 4, Neural networks for attention to internal and external sensory cues in ASD (4.7%); 5, Mapping thalamocortical networks across development in ASD (1.4%). Studies 4 and 5 originated from the same research group and utilized the same recruitment and scan protocols, so we combined them when making pairs of ASD-TD subjects. All studies assessed TD subjects with standardized cognitive and behavioral instruments and excluded any subjects with structural brain injury, intellectual disability, developmental delay, a family history of ASD in a first-degree relative, or any neurological diagnosis. The subjects scanned through these studies have been described previously [43–46]. While some subjects were scanned in more than one study, or were scanned more than once in a single study, we included only one timepoint for each subject. All subjects assessed in this study signed written informed consent prior to participation in the original research and consented to sharing their research data through the NDA. The Institutional Review Board of the University of Texas Southwestern at Dallas approved our secondary analyses of this shared data. All experiments were conducted in accordance with the principles of the Declaration of Helsinki.

We assembled pairs of subjects, matching subjects with ASD and TD controls based on sex, age, imaging protocol, and by self-reported race. For subjects younger than 240 months we matched within 12 months; between 241–360 months, we matched within 24 months; and older than 360 months, we matched within 48 months. We generated pairs within an imaging protocol (intra-study matching) wherever possible; we generated between-study pairs only for subjects that had no match within their study of origin. Since studies 4 and 5 originated from the same research group and shared an imaging protocol, we matched within the group of both studies. When experimental pairs included subjects from different studies, we attempted to equalize the number of cross-study contributions between ASD and TD cohorts; if study X supplied an ASD subject in a cross-study pair, we tried to match a TD subject from study X in a separate cross-study pair. Self-identified racial descriptions included Asian, Black, Native American, White, and more than one race. When pairs could be matched for some but not all criteria, we followed this hierarchy: sex (obligatory match) > minimizing age difference > originating study > self-identified race. Handedness and IQ scores were not supplied for a sizeable fraction of our cohort, and thus was not included as a matching requirement.

All subjects in our ASD cohort were assessed with the Autism Diagnostic Observation Schedule (ADOS), modules 3 or 4, which require intact fluent speech. ADOS scores reflect clinician ratings of observed social, communication and repetitive behaviors; higher scores indicate a greater degree of social-communication and behavioral impairments that are more common among individuals with ASD. Each source study categorized ADOS scores into high-elevated or mid-elevated, which the studies labeled as “autism” or “autism spectrum disorder.” However, granular ADOS results were not available for all subjects. Therefore, we were unable to assess the influence of individual ADOS domains on striatal compartment volume. To avoid any confusion with our ASD cohort (which includes all subjects whose scores met criteria for the diagnosis of autism or autism spectrum disorder), we refer to the diagnostic labels from the originating studies as ADOS-high and ADOS-mid.

Though granular ADOS data was not available for all subjects in our matched cohorts (213 pairs), we assembled a cohort of ASD subjects for whom full ADOS data was available. This cohort included all ASD subjects from our five originating studies, not solely those in our 213 matched pairs. 160 subjects had ADOS data, and 155 of those were also present in our 213 matched pairs (72.8% of the matched-pairs cohort was represented in this second cohort of ASD subjects). ADOS scores for these subjects included Modules 3 and 4, and utilized both ADOS (pre-2012) and ADOS-2. Since these versions are configured and scaled differently, we converted all ADOS scores into Calibrated Severity Scores (CSS) [47–50]. We assessed matrix-like volume vs. CSS in this larger, non-matched ASD cohort.

MRI Acquisition, DTI processing, and Anatomical Segmentation of ROIs can be found in our Supplemental Methods.

### Parcellating the striatum into striosome- and matrix-like compartments

We added five matrix-favoring regions (supplementary motor area, primary motor cortex, primary sensory cortex (Brodmann areas 1–3), the combined ventrolateral (VLc and VPLo) thalamic nuclei, and the globus pallidus interna) to create a composite matrix-favoring target mask. Similarly, we generated a composite striosome-favoring target mask by adding five striosome-favoring regions (posterior orbitofrontal cortex, basal operculum, anterior insula, basolateral amygdala, and mediodorsal thalamus). These composite target masks were necessary to account for two features identified by prior tract tracing studies in animals: each region is biased in its projections toward one compartment, but includes a blend of matrix-projecting and striosome-projecting neurons [51, 52]; preference for one striatal compartment may be limited to one part of the striatum, with loss of specificity in other parts of the striatum [37]. We aimed to reduce any such off-target influences by combining the strength of connectivity across the group of compartment-favoring regions.

We performed classification targets tractography (CTT) in native space, utilizing the FSL tool *probtrackx2* [53], with each hemistriatum as seed and the ipsilateral matrix-favoring and striosome-favoring composite masks as target. We utilized a midline exclusion mask in the sagittal plane to ensure that projections remained ipsilateral; we ran each hemisphere separately. Connection probability was corrected for path length. We utilized the following *probtrackx2* parameters: curvature threshold = 0.2; steplength = 0.5 mm; number of samples = 5,000; number of steps per sample = 2,000. Each voxel, in each subject, for each hemistriatum, was assigned a probability (0.0–1.0) of tractographic connectivity with either matrix-favoring or striosome-favoring brain regions, thus generating a probability map for each subject’s hemistriatum. Voxels with biased probability of connection (≥ 0.55) were defined as striosome-like or matrix-like. We describe these voxels as “-like” for two reasons: first, the diameter of the human striosome in coronal sections ranges from 0.5–1.25 mm (based on histology presented by Graybiel & Ragsdale [54] and Holt et al. [55]), so every diffusion voxel (2 mm isotropic) has the potential to include both striosome and matrix elements; second, parcellation identifies voxels whose pattern of structural connectivity matches the properties of matrix or striosome identified in animals, but this should not be conflated with direct identification through immunohistochemical staining, the gold standard for striatal compartment segregation. Selecting voxels with higher connection probability increased the likelihood that a given voxel A) included matrix in isolation, or B) included predominantly, though not entirely, striosome.

### Volumetry of striatal compartments

We quantified the volume of each compartment-like parcellation in native space at two probability thresholds: 0.95 and 0.55 (the minimum classification threshold). We also quantified indeterminate voxels, those with connection probability of 0.45–0.55, that were not classified into either compartment. We measured compartment-like volumes for the whole striatum, as well as separate measures for compartment-like volume within caudate or putamen. Finally, we performed a histogram analysis of the matrix-like probability distribution (0–1.0) using the FSL command *fslstats* with 100 bins. Thought experiments, described in our Results section, led us to reason that testing for compartment-specific group differences at the uppermost (top five bins) of each probability distribution could distinguish between hypothesized tissue-level causes of volume abnormalities.

### Defining striatal compartment masks for subsequent quantification steps

Probabilistic tractography may be biased by size differences between target masks; to accurately quantify the connectivity of matrix-like and striosome-like voxels, we generated compartment-specific striatal masks that were equal in size for each hemisphere. We selected from among biased voxels (probability of connection ≥ 0.55, ensuring that each candidate voxel was matrix-like or striosome-like), starting at the uppermost probability of biased connection (1.0) and accepting voxels until we reached a preset volume threshold. We thus ensured that each striatal compartment was represented by an equal-sized mask made up of its most-discriminating voxels. We established a volume threshold of 83 native space voxels by starting with the volume of each hemistriatum in the MNI_152_1mm template brain, eliminating the volume that would make up the median 1.5SD in a normally distributed volume, and splitting the remaining volume across two tails of the distribution. Masks with 83 native space voxels therefore represent the fraction of the probability distribution 1.5SD above the mean for each striatal compartment.

Within an individual and hemisphere, the volume of matrix-like and striosome-like masks was always matched. For some participants, connectivity bias was non-normally distributed. In participants with strong bias toward one category of extra-striatal targets, it was occasionally impossible to identify 83 voxels that passed the probability threshold (≥ 0.55). In those circumstances, striatal masks for both compartments were set to N, the number of voxels with probability ≥ 0.55. The mean volume of these masks was matched for the matrix-like and striosome-like distributions, and was equal in ASD and control subjects (82.5 voxels for each). We used these equal-volume striatal masks as the targets for subsequent rounds of tractography. Given that matrix and striosome are not distributed randomly in the striatum (striosome is enriched rostrally, medially, and ventrally [54, 56–58]), we assessed the Cartesian position of each voxel in these equal-volume masks (138,347 voxels) relative to the centroid of the nucleus it occupied (left or right hemisphere, caudate or putamen). These measures allowed us to gauge the accuracy of our parcellations as matrix-like or striosome-like.

### Evaluating potential alternate explanations for abnormal striatal compartment volume

Our striatal parcellation method is dependent on differential structural connectivity. Differing tissue volume of matrix and striosome is one potential reason we might detect abnormal matrix-like or striosome-like volume. However, any factor that has differential influence on matrix-favoring or striosome-favoring streamlines could skew striatal parcellation towards one compartment. We measured the volume of the striatum, and of the matrix-favoring and striosome-favoring bait masks, in native space for each individual; a group-level change in these bait regions could skew parcellation. To investigate the potential for abnormal striatal organization to influence parcellation, we extracted fractional anisotropy (FA) in native space using each subject’s matrix-like and striosome-like striatal masks.

Next, we performed two iterations of quantitative probabilistic tractography to assess for any such distorting factors. First, we seeded the matrix-favoring or striosome-favoring extra-striate masks (those we had utilized as bait for striatal parcellation) and quantified the streamlines that contacted the ipsilateral striatum (*probtrackx2* output: waytotal). Seeding 50 streamlines/voxel provided sufficient streamline density to quantify connectivity for these large-volume masks. Tractography parameters otherwise matched those described above. Second, we seeded the 1.5SD matrix-like and striosome-like striatal masks with 5000 streamlines/voxel and quantified all ipsilateral extra-striate projections (waytotal). We further characterized compartment-specific structural connectivity by assessing the volume of overlap (Dice similarity coefficient, DSC) in streamline volumes seeded by matrix-like or striosome-like voxels, as follows:

$$\frac{2\times {(Volume}_{Projection from Striosome-like Seed }\cap {Volume}_{Projection from Matrix-like Seed})}{{Volume}_{Projection from Sriosome-like Seed}+ {Volume}_{Projection from Matrix-like Seed}}$$

We calculated DSC in native space for each individual and hemisphere, with an amplitude threshold set to retain the uppermost 75% of voxels.

### Somatotopic organization of striatal projections

Prior tract tracing studies observed a somatotopic organization of cortico-striate projections, with a given region projecting to some, but not all, parts of matrix or striosome [59, 60]. We sought evidence of this somatotopic organization in human compartment-like voxels by comparing probability maps between two conditions: when segmented using all of the bait regions (5 matrix-favoring, 5 striosome-favoring, none left out), and when segmented using only N-1 regions (9 regions, one left out). The matrix-like probability distribution (P) unique to region A, distinct from that of regions B-J, follows the formula PN-1(A-J)—PN-1(B-J) = PN-1A; comparing probability distributions between two starting conditions (ten bait regions vs. nine bait regions) defines the contribution of the tenth, left-out region. For striosome-favoring bait regions we utilized the inverse of the formula noted above – the sites where striosome-favoring regions had the greatest influence on matrix-like connection probability. For each hemisphere, and for each of our 10 “bait” regions, the average probability distribution from our 213 control subjects served as the source of our somatotopic maps. We selected for the most highly-enriched voxels using a probability threshold (using the FSL tool *fslmaths* -thrP) to retain voxels whose connectivity was at or above the 5th percentile. It is notable that by selecting for voxels that have the strongest contribution from a single region, this method underweights voxels in which strong connectivity is contributed by multiple regions.

### Statistical analyses

We carried out statistical testing using Stata (StataCorp, 2023, Stata Statistical Software: Release 18.0. College Station, TX). We performed multiple linear regression to identify demographic and experimental factors that influenced matrix-like volume, defined by probability of connecting to matrix-favoring targets at P ≥ 0.95: age, sex, self-identified race, diagnosis (TD, ADOS-high or ADOS-mid), originating study, the hemisphere assessed, and the volume of the diffusion-space striatal mask utilized to seed tractography. Age and striatal volume were not independent, so we elected to include the variable with greater impact on matrix-like volume. In parallel regressions, differing only in the inclusion of age or striatal volume, striatal volume had a substantially greater influence on matrix-like volume than age (t-statistics of 7.0 and 1.8, respectively). Therefore, we did not include age in subsequent regressions. Four variables significantly influenced matrix-like volume (diagnosis, originating study, hemisphere, and striatal volume). For all variables that significantly influenced matrix-like volume, we performed a secondary linear regression assessing only those variables and their interactions. Hemispheric differences made up only 1.5% of the total striatal volume, the smallest contribution of these four variables. Therefore, for subsequent comparisons we opted to not segregate data based on hemisphere of origin. In the cohort of ASD individuals with ADOS scores (72.8% of the paired cohort), we performed a distinct linear regression to assess the relationship between matrix-like volume and CSS, a metric used to combine ADOS data across modules and versions.

We utilized four distinct families of t-tests (two-tailed, paired samples) to assess the following experimental questions: i) is there a compartment-specific difference in volume between ASD and TD, and ii) are those differences present in both caudate and putamen; iii) in what parts of the probability distribution do we find any differences; iv) do compartment-specific volume differences localize to particular somatotopic zones; v) are differences in compartment-specific volume attributable to specific bait regions? We controlled the false discovery rate (FDR) within each family of tests using the method of Benjamini and Hochberg [61]. First, we compared the TD and ASD cohorts for group-level differences in compartment-specific volume (matrix-like volume, striosome-like volume), and then for inter-compartment difference within each subject. We assessed volume at two probability thresholds (0.95, 0.55). We then tested whether volume differences in the caudate and putamen (the two regions that make up the striatum) matched the volume differences identified in the whole striatum (one-tailed, paired samples, at the same probability thresholds). Our FDR-corrected significance threshold for this family of tests was *p* < 0.029. Second, we quantified compartment-specific volume at the uppermost ends (top five bins) of the striosome-like and matrix-like probability distributions. Our FDR-corrected significance threshold for these tests was *p* < 0.04. Third, we measured for extra-striate compartment bias in each of our 10 bait regions using our N-1 striatal parcellations as targets and the left-out region as seed. Our FDR-corrected significance threshold for this family of tests was *p* < 5.0 × 10^–3^. Fourth, we identified the intra-striate somatotopic zone in which each bait region was the primary driver of connectivity. Within each zone we assessed the volume of matrix-like voxels, comparing TD and ASD cohorts. Our FDR-corrected significance threshold for this family of tests was *p* < 0.04.

We performed two types of post-hoc experiments to check the validity of our primary findings. First, we utilized t-tests to assess potential alternate explanations for our compartment-specific volume measures: abnormal tissue-level structural organization (FA); abnormalities in general structural connectivity (streamline counts). Second, we established a priori that for all bait regions whose somatotopic zones had ASD-specific increases in matrix-like volume, we would utilize quantitative tractography (cortical seed to striatal compartment targets) to characterize regional abnormalities as striate-alone or combined striate and extra-striate. This resulted in seven t-tests comparing streamline counts (paired samples, two tailed, ASD vs. TD). The Benjamini–Hochberg corrected significance threshold for this family of tests was *p* < 7.1 × 10^–3^.

## Results

### Experimental cohorts

We assembled 213 experimental pairs, comprised of one subject with ASD and one TD control, that were matched, to the best of our ability, for sex, age, self-identified race, and type of dMRI protocol. All pairs were matched for sex (30.0% female). 212 pairs were matched for age; one pair (mean age: 181 months) was separated by 13 months instead of the target, ≤ 12 months. The mean within-pair age difference was 5.2 months. Our subjects ranged from 78–508 months (mean: 182 months). 79% of our pairs were matched for self-identified race. 81% of our subjects self-identified as White, a category that included individuals who identified as Hispanic/Latino. 89% of pairs were matched within the same study and thus were imaged with identical dMRI protocols. A further 4.7% of subjects were paired between studies but were balanced by a pair with the opposite pattern (pair_StudyA-StudyB balanced by pair_StudyB-StudyA). Thus 93.4% of our subjects were matched for dMRI protocol. Within our ASD cohort, 166 subjects (77.9%) were in the ADOS-high category, and 47 subjects (22.1%) were in the ADOS-mid category. We also assembled a distinct cohort of subjects with ADOS scores (rather than relying on the assigned diagnostic category from the originating study) and converted those scores to CSS for comparison across versions and modules of ADOS. This cohort included 160 subjects with a mean CSS of 7.1 (SEM: 0.096; range: 3–10).

### The intrastriate location of compartment-like voxels

We measured the position of each voxel in our matched-volume highly-biased (matrix-like or striosome-like) voxels. The mean location of striosome-like voxels was 0.4 mm more medial (*p* < 1.0 × 10^–100^), 5.0 mm more rostral (p < 1.0 × 10^–100^), and 3.7 mm more ventral (*p* < 1.0 × 10^–100^) than the mean location of matrix-like voxels. This matched the expected location biases or striosome established through human immunohistochemistry [54, 55]. Note that since individual voxels were assessed relative to the centroid of either caudate or putamen, the relative positioning of each nucleus within the hemisphere did not drive these differences in location. The locations of matrix-like and striosome-like voxels did not differ, on average, between ASD and TD cohorts. The root-mean-square distance between mean ASD and mean TD voxel location was 0.15 mm for matrix-like voxels, and 0.13 mm for striosome-like voxels.

### Striatal compartment volume, ASD vs. TD

**Fig. 1:**
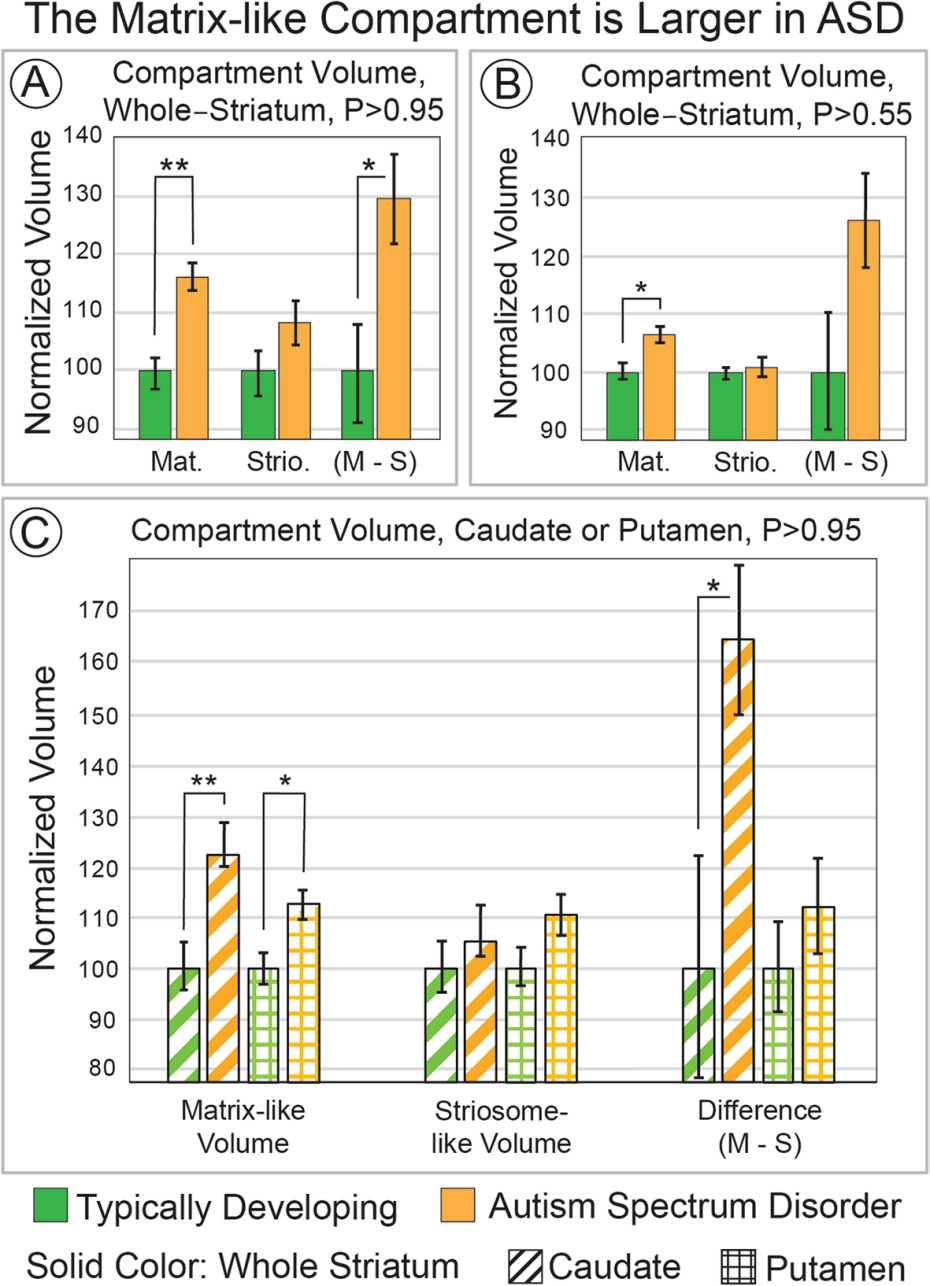
The Matrix-like Compartment is Larger in ASD. Striatal voxels that had biased structural connectivity towards matrix-favoring regions (matrix-like voxels) were identified in greater abundance in autism spectrum disorder (ASD) than in matched typically developing controls. This volume expansion was present in highly-biased voxels (panel **A**, probability threshold ≥ 0.95) and minimally-biased voxels (panel **B**, probability threshold ≥ 0.55). In panel **C**, we assessed compartment-specific volume in the two regions that make up the striatum, the caudate and putamen (identified by stripes or checks, respectively). The matrix-like compartment was expanded in both caudate and putamen, but this increase was larger in the caudate. In addition to group-average comparisons, within-individual differences in compartment volume (M–S; matrix volume—striosome volume) suggest that the expansion in matrix-like voxels in ASD is present in individuals with ASD, not solely by group-level differences (**A**, **B**, **C**). Striosome-like voxels did not significantly differ between ASD and controls at any probability threshold, or for any region, even for significance thresholds not adjusted for multiple comparisons (smallest *p*-value, *p* ≤ 0.16). **, *p* ≤ 5.6 × 10^–5^; *, *p* ≤ 5.6 × 10^–3^

We extracted the volume of highly-biased voxels (*P* ≥ 95%, 1.96 standard deviations (SD) above the mean) toward matrix-favoring or striosome-favoring regions (test: hypothesis i). In ASD, the matrix-like compartment was expanded by 16.5% (mean, 245 vs. 211 voxels; *p* = 9.5 × 10^–6^; Fig. 1A), while the striosome-like compartment was not significantly different in ASD and TD (mean, 146 vs. 134 voxels; *p* = 0.10). The within-subject difference in compartment-specific volume (matrix minus striosome) was greater in ASD as well (+ 29.9%; 99 voxels in ASD vs. 76 voxels in TD; *p* = 0.016). We also assessed compartment-specific volume at P ≥ 0.55, the lowest margin for defining compartment-specific connectivity. Matrix-like volume was expanded in ASD at this minimum probability threshold (mean, 485 vs. 457 voxels, + 6.3%; p ≤ 9.5 × 10^–4^; Fig. 1B) while striosome-like volume was not different (ASD + 0.71%; *p* = 0.74). At this minimum threshold, the within-subject differences in.

compartment volume were larger in ASD, though not a significant degree (mean, 126 vs. 100 voxels, + 26.1%; p = 0.047). The expansion in matrix-like voxels was substantially larger in ADOS-high subjects (+ 19.6%; p = 1.2 × 10^–6^) than in ADOS-mid subjects (+ 5.3%; *p* = 0.38).

**Fig. 2:**
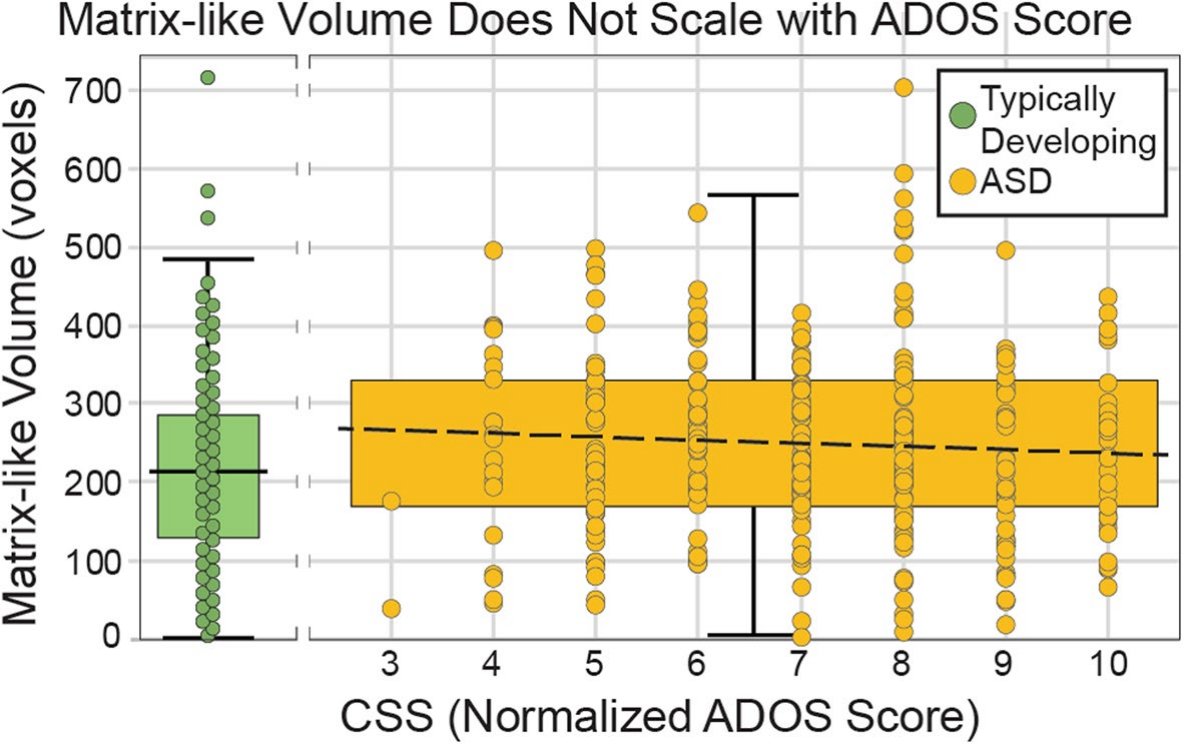
Matrix-like volume vs. ADOS score. In ASD (orange), the Calibrated Severity Score (CSS, which normalizes ADOS scores between versions and modules) had little influence on matrix-like volume. While mean matrix-like volume (*P* ≥ 0.95) was elevated relative to typically-developing (TD, green) controls, this elevation did not scale with CSS. The dashed line in the elevated ASD box plot (orange) represents the regression coefficient for this cohort (R^2^ = 0.0027). Note that TD volume measures represent the same mean data shown in Fig. 1. While all subjects were assessed with ADOS, individual scores were provided for few TD subjects. Therefore, TD subjects are illustrated here without CSS scores

In a cohort of individuals with complete ADOS data (largely overlapping, but not synonymous with the matched ASD-TD pairs noted above), subjects with elevated CSS (range: 3–10) had elevated matrix-like volume (highly-biased voxels, P ≥ 95%; mean: 248 voxels; SEM: 6.5; Fig. 2). However, increased matrix-like volume did not covary with CSS (coefficient: −3.5; R^2^ = 0.0027, F_(1, 318)_ = 0.86, *p* = 0.35, 95% CI [−11.0,4.0]) – the category designation of “autism” was associated with increased matrix-like volume, independent of CSS. That is, group-wise comparisons of matrix-like volume were significantly different but the correlation using continuous variables was not.

Matrix-like volume was larger in ASD in both the caudate and putamen, but the expansion in the caudate was double that found in the putamen (test: hypothesis ii; caudate: + 24.0%, *p* = 8.2 × 10^–5^; putamen: + 10.3%, *p* = 8.1 × 10^–3^; Fig. 1C). The within-subject difference in compartment volume was significant in the caudate (ASD, + 64.2%; p = 0.017), but not for putamen (ASD, + 11.7%; *p* = 0.36). Striosome-like volume did not differ between ASD and controls for either caudate or putamen.

The expansion of the matrix-like compartment occurred within a larger striatum. Native-space striatal masks were 3.5% larger in ASD (926 vs. 895 voxels, *p* = 3.4 × 10^–3^). This expansion was similar in the left and right hemispheres (+ 3.2% and + 3.9%, respectively). In contrast, the volume of the extra-striate bait regions we used to parcellate the striatum did not differ between ASD and TD (matrix-favoring: + 0.068% in ASD, *p* = 0.93; striosome-favoring: + 0.32% in ASD, *p* = 0.73).

**Fig. 3:**
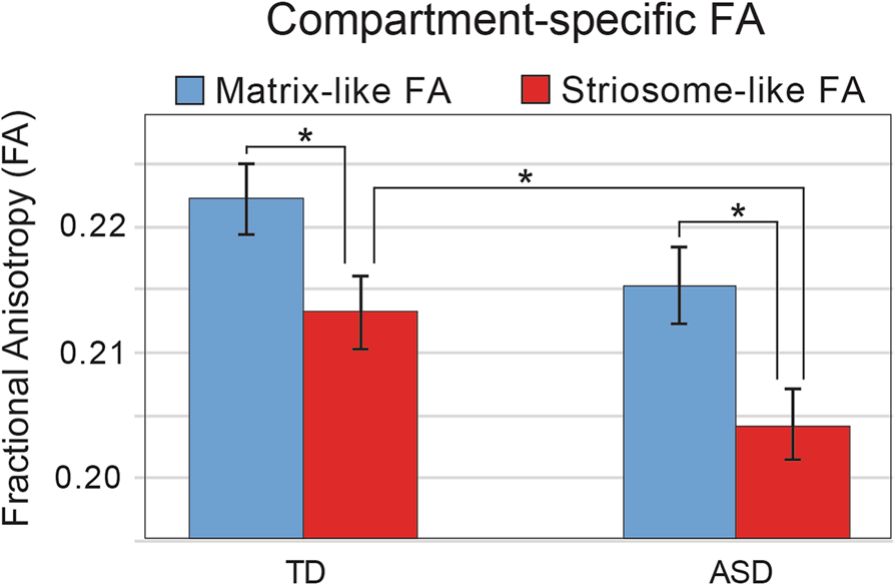
Compartment-specific Fractional Anisotropy. Fractional anisotropy (FA) is higher in matrix-like voxels than in striosome-like voxels in both typically developing (TD) controls and in individuals with autism spectrum disorder (ASD). While FA was significantly lower in the striosome-like voxels of ASD subjects, there was no difference in the FA of matrix-like voxels between TD and ASD. *, *p* < 0.0378

Our parcellation technique is based on differential connectivity. Therefore, factors that skew connectivity might lead to an increase in detection of matrix-like voxels in ASD without a true tissue-level expansion in matrix volume. We previously demonstrated that selecting striatal voxels based on FA, rather than selecting for precise connectivity, strongly biases towards a matrix-like pattern of connectivity [58]. We also demonstrated, in healthy subjects, that matrix-like voxels have significantly higher mean FA than striosome-like voxels [58]. Our TD cohort had a highly similar pattern, with FA 4.2% higher in matrix-like voxels than in striosome-like voxels (0.222 vs. 0.213; *p* = 4.1 × 10^–4^; Fig. 3). In ASD, FA was 5.4% higher in matrix-like voxels (0.215 vs 0.204; *p* = 5.5 × 10^–6^). The ratio of FA between the compartments (matrix-like:striosome-like voxels) did not differ between ASD and TD (*p* = 0.71). Therefore, it is unlikely that differences in the architecture of matrix-like voxels (as measured by FA) drove increased parcellation of matrix-like voxels. FA in matrix-like voxels did not significantly differ between the cohorts (ASD, −3.2%, NS), while FA in striosome-like voxels was significantly lower in ASD subjects than in TD subjects (ASD, −4.4%, *p* = 0.015).

Identifying a single voxel as matrix-like or striosome-like depends on the relative abundance of streamlines seeded by matrix-favoring vs. striosome-favoring bait regions. We considered the possibility that extra-striate feature of the ASD brain might bias connectivity such that matrix-favoring streamlines were enriched, without a change in the nature of striatal tissue. Increasing the probability that a voxel will parcellate as matrix-like voxels, with no change in the volume of striosome-like voxels, would by necessity pull voxels from the indeterminate pool (0.45 ≤ *P* ≤ 0.55) into the matrix-like distribution. We found that indeterminate volume did not differ between ASD and TD cohorts (mean of 38.8 vs 41.2 voxels, respectively; p = 0.11), suggesting that the expansion of matrix-like volume in ASD does not result primarily from better tissue parcellation in ASD.

Next, we tested the hypothesis that the increase in matrix-like voxels in ASD is secondary to projections to the striatum, rather than the tissue composition of the striatum. We assessed differences in connectivity in two scenarios. First, we measured the number of completed streamlines following tractography with our extra-striate bait regions as seeds and the whole striatum (not selective for matrix-like or striosome-like voxels) as an obligatory waypoint. Streamlines seeded by both striosome-favoring and matrix-favoring bait regions were reduced in ASD relative to TD (matrix-favoring: ASD, 10.6% less, *p* = 2.9 × 10^–2^; striosome-favoring: ASD, 12.1% less, *p* = 6.4 × 10^–3^). The ratio of total matrix-favoring:striosome-favoring streamlines within each subject was not significantly different between TD and ASD (*p* = 0.17). This suggests that the expansion in matrix-like volume we identified was not primarily the result of differences in the structural connectivity of our extra-striate bait regions.

**Fig. 4:**
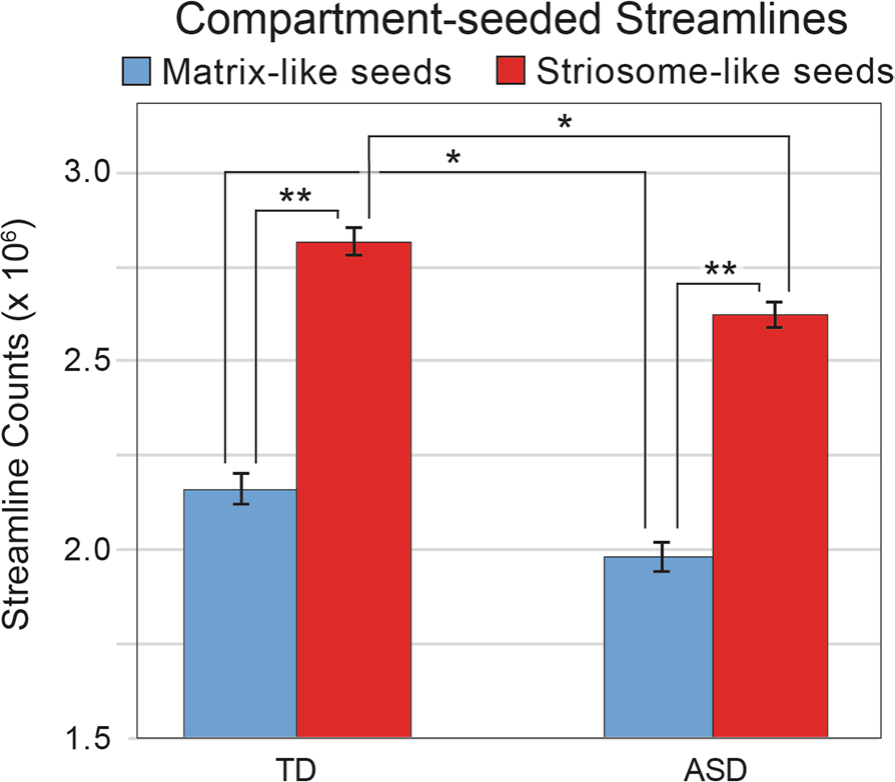
Quantitative structural connectivity with compartment-like voxels. In both typically developing (TD) controls and autism spectrum disorder (ASD), striosome-like seed voxels (red) propagate more streamlines than matrix-like seed voxels (blue), despite the fact that seeds are matched for volume and project to identical targets under identical tractographic conditions. However, ASD subjects propagate significantly fewer streamlines than TD subjects for both striosome-like and matrix-like seeds. *, *p* < 5 × 10^–4^; **, *p* < 5 × 10^–67^

Second, we measured the number of completed streamlines following tractography that set either matrix-like or striosome-like voxels as seeds and all ipsilateral extra-striate gray matter as an obligatory waypoint. In both matrix-seeded and striosome-seeded streamline volumes, fewer streamlines completed tractography in ASD (matrix-like: ASD 9.1% less, *p* = 4.4 × 10^–4^; striosome-like: ASD 7.4% less, p = 3.5 × 10^–5^; Fig. 4). In both ASD and TD, streamlines seeded by striosome-like voxels were more likely to reach gray matter targets than streamlines seeded by matrix-like voxels (ASD: striosome 32.3% more, *p* = 4.1 × 10^–67^; TD: striosome 30.3% more, *p* = 2.6 × 10^–72^). For individual subjects, striosome-seeded streamlines were more abundant than their matrix-seeded streamlines in 87.1% of TD subjects and 84.0% of ASD subjects. Notably, we previously found this striosome-dominant pattern of connectivity in a different neuroimaging cohort [58]. The matrix-seeded and striosome-seeded mean streamline distributions were highly segregated, with DSCs of 1.9% and 3.1% (ASD and TD, respectively). It is notable that these comparisons of matrix-seeded and striosome-seeded streamlines were identical in their tractography parameters; they utilized matrix-like and striosome-like seed volumes whose volume was identical within each individual and hemisphere, they targeted the same whole-hemisphere gray matter mask, and each iteration of tractography seeded the same number of streamlines per seed voxel. These marked differences in the distribution of streamline bundles between matrix-like and striosome-like voxels did not differ between ASD and TD (*p* = 0.73).

### Tissue-level origins of increased matrix-like volume

**Fig. 5:**
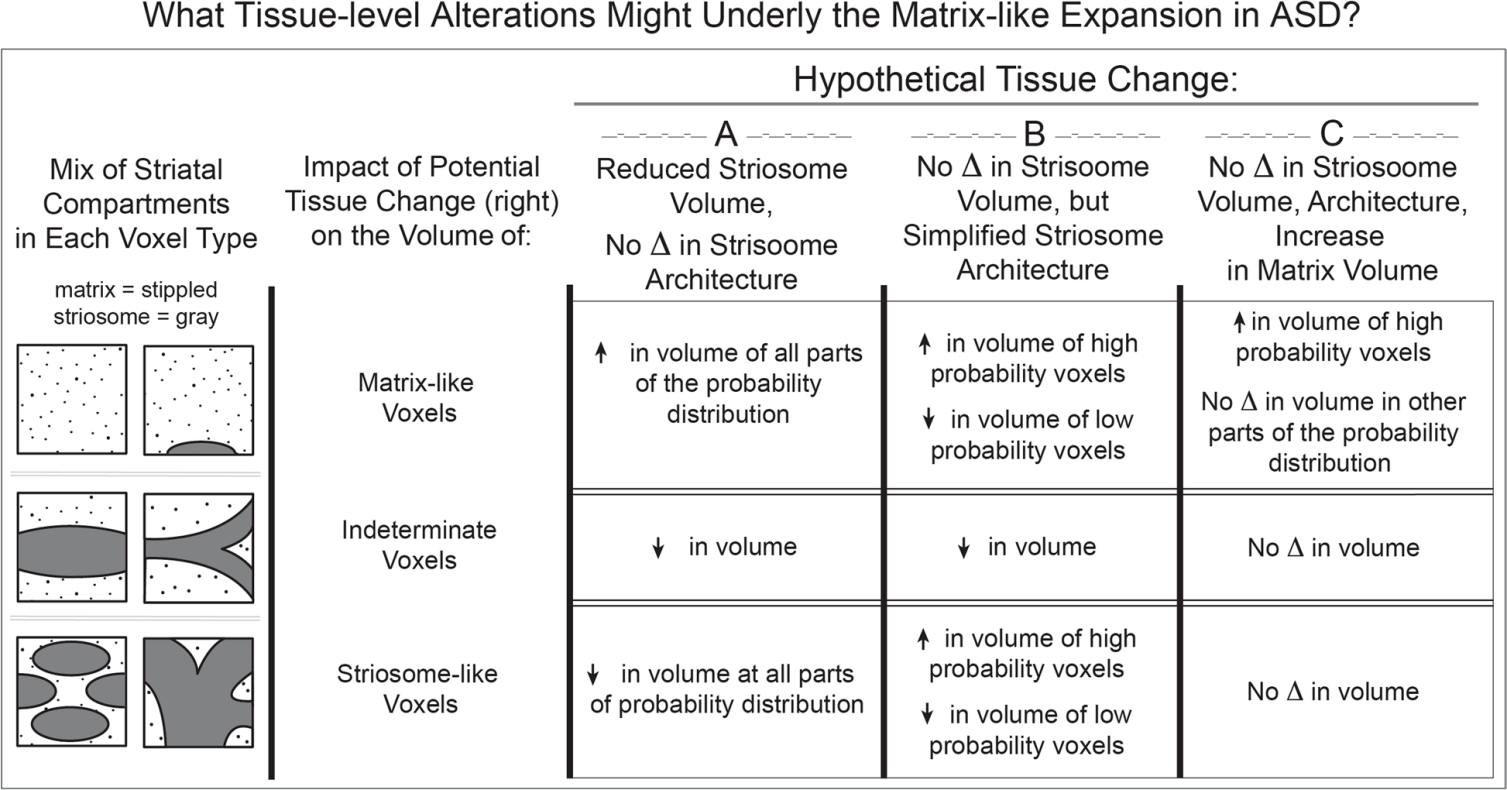
Potential Tissue Alterations Underlying Increased Matrix-like Volume. Each diffusion voxel (resolution = 2 mm isotropic) has the potential to include both matrix and striosome. The character of the tissue included in each voxel determines whether it will be parcellated as matrix-like, striosome-like, or indeterminate. Three potential changes to striatal tissue – a decrease in striosome numbers or projections to the striosome (**A**); a simplification of striosomal branching and complexity (architecture, **B**); an increase in matrix volume with no change in striosome (**C**) – can explain the increase in matrix-like volume we detected in ASD. Granular measurements of compartment bias throughout the probability distribution (histogram analysis, Fig. 6) allow us to infer the nature of the tissue abnormality in ASD that leads to an expansion in matrix-like volume

We aimed to identify the type of tissue-level change responsible for the expansion in matrix-like volume (Fig. 1). Since matrix-like voxels were defined by differential connectivity, this volume expansion could result from increases in matrix connectivity, reductions in striosome connectivity, or a combination of both changes. As noted above, we found cohort-specific reductions in completed streamlines (ASD vs. TD) but no compartment-specific differences (matrix-like vs. striosome-like) in global measures of structural connectivity in either our bait regions or in our parcellated striatal voxels. This suggested that the ASD-specific changes in matrix-like volume resulted primarily from striatal abnormalities, not to abnormalities in striatal afferents. Three potential tissue-level striatal abnormalities could explain our imaging findings: a decrease in striosome volume, with no change in striosomal architecture (the pattern of striosomal branching); a simplification of the striosomal architecture such that the same volume of striosomal tissue is concentrated in a smaller number of voxels (thicker striosome “tubes,” fewer striosome branches); an expansion in the volume of the matrix with no change in the volume or architecture of the striosome. Though these tissue abnormalities are not mutually exclusive, considering the impact of each abnormality in isolation, and considering how each change would be sampled at the scale of our diffusion voxels, allowed us to predict and assess for three distinct patterns of change in striatal compartment volume (Fig. 5).

The striosome is a highly-branched, labyrinthine structure [62] that is largely surrounded by matrix. With diameters of approximately 0.5–1.25 mm in the coronal plane [54, 55], the branches of the human striosome are smaller than our diffusion voxels (2 mm isotropic). Therefore, each striatal voxel may include matrix in isolation or a blend of matrix and striosomal tissue – but never striosome in isolation. At a particular intra-voxel ratio of matrix:striosome tissue (the precise limits of which are the subject of ongoing investigation), that voxel’s connectivity will be indeterminate. On either side of that ratio, connectivity will be biased towards matrix-favoring or striosome-favoring targets. Since voxel position is distributed randomly with respect to striosome location, some voxels will be centered on a striosomal trunk, but many voxels will intersect with branchpoints or cleave the striosome obliquely (Fig. 5, left-most column). While a voxel may reach probability threshold (and thus be parcellated as striosome-like) by including a “direct hit” or by sampling parts of multiple striosomal branches, each of the hypothesized changes to tissue-level composition will shift the likelihood of a direct hit vs. oblique sampling. Each theorized striatal tissue abnormality will therefore produce distinct alterations to the compartment-specific probability distributions.

Within striatal tissue, a reduction in striosome volume, with no change in striosomal architecture (Fig. 5A), would reduce the number of voxels with all types of striosome-like connectivity, both “direct hits” and off-center sampling. With this hypothetical tissue change, all types of striosome sampling – direct hits, oblique cuts, and glancing contacts – would be less common. Therefore, a histogram of connection probabilities would shift toward matrix-like bias throughout the probability distribution. Fewer voxels would include a balanced mix of matrix and striosome tissue, reducing the number of indeterminate voxels.

If striosome volume is unchanged but the architecture of striosome branches is modified (Fig. 5B), different impacts on striatal parcellation emerge. Organizing striosomal volume into fewer, thicker tubules (imagine a net with larger openings but thicker cords connecting each knot) would increase the number of high-probability voxels (since thicker striosome tubules would occupy a larger fraction of “direct hit” voxels). With fewer tubules, fewer voxels would make oblique cuts through the striosome, reducing the number of both low-probability striosome-like voxels and indeterminate voxels. Likewise, thicker, less-abundant striosomal branches would result in a larger number of voxels that included only matrix, as well as a reduction in the volume of obliquely sampled striosome within matrix-like voxels. This hypothetical tissue change would therefore shift volume from the center of the probability distribution to both ends, increasing the volume of both highly biased matrix-like and striosome-like voxels and reducing the volume of low bias and indeterminate voxels.

Increased matrix-like volume could also result from an expansion in the volume of matrix tissue, with no change in the striosome (Fig. 5C). The striosome is surrounded by matrix. Therefore, the number of voxels that sample a mixture of matrix and striosomal tissue is determined by the surface area of the striosomal arborization. If one holds the volume and architecture of the striosome constant, adding additional matrix volume will not increase the amount of overlap between the compartments – adding matrix tissue directly adjacent to a striosome would simply displace other matrix tissue, increasing the number of voxels that sample matrix in isolation. This hypothetical expansion in matrix tissue, with no change in the striosomal compartment, would lead to a selective increase in volume at the uppermost end of the matrix-like probability distribution with no changes in volume at other parts of the probability distribution. Since this increase in matrix tissue would not increase the amount of within-voxel averaging of the striatal compartments, the striosome-like probability distribution, and the volume of indeterminate voxels, would be unchanged.

### Histogram analysis of compartment-specific probability

**Fig. 6:**
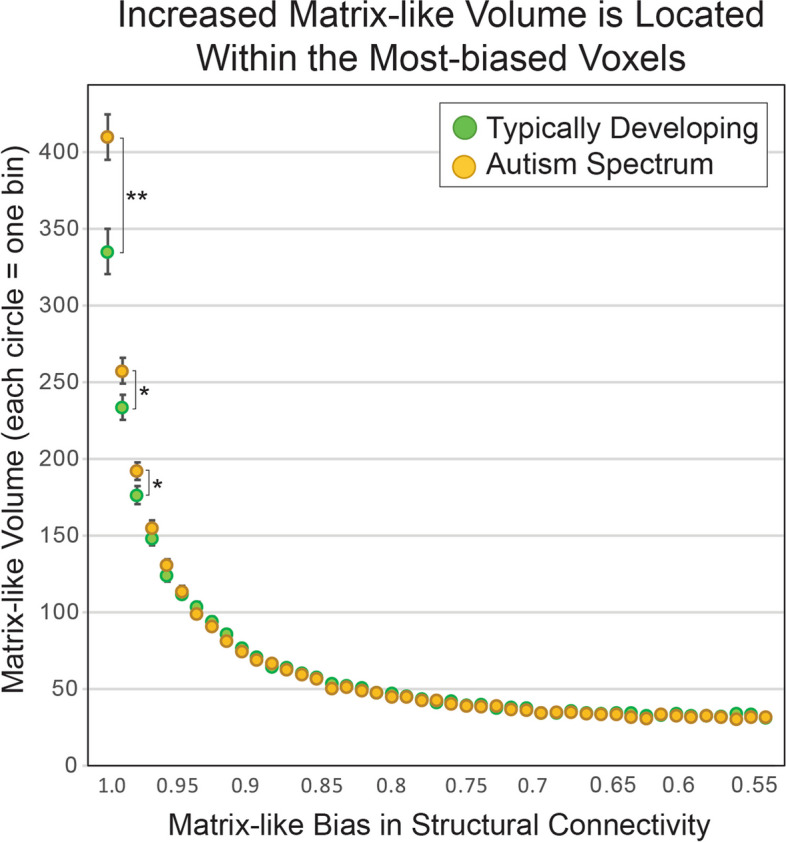
Expansion in Matrix-Like Volume Results from a Selective Increase in High-Bias Voxels. Increased matrix-like volume could result from multiple tissue-level changes to matrix or striosome. Histogram analyses can identify the type of voxels (high- vs. low-bias; matrix vs. striosome) that were changed in autism spectrum disorder (ASD) relative to Typically Developing (TD) controls, and thus allowed us to evaluate the potential tissue-level changes hypothesized in Fig. 5. We performed histogram analyses on the matrix-like and striosome-like probability distributions (1.0 to 0.55 for each), quantifying the voxels with compartment-specific connectivity in 0.01 unit bins (each circle = one bin, 45 total bins). Matrix- and striosome-like histograms followed similar patterns, but matrix-like volume diverged in ASD at the highest probability bins (left-to-right = high-to-low probability); below probabilities of approximately 0.95, matrix-like volume was indistinguishable in ASD and TD. Striosome-like volume did not differ between ASD and TD at any part of the probability distribution (data not shown); expansion in matrix-like volume cannot be attributed to a spurious overcount of matrix-like voxels due to a decrease in striosome-favoring connectivity. **, *p* = 2.5 × 10^–4^; *, *p* ≤ 0.02

We utilized a histogram analysis (test: hypothesis iii; Fig. 6) to differentiate between the tissue-level changes hypothesized above (Fig. 5). We sampled compartment-specific volume along the probability distribution in 0.01-unit increments from 1.0 to 0.55. The expansion in matrix-like volume in ASD occurred entirely in the highest probability (most biased) voxels (Fig. 6); the uppermost three bins contributed 88.0%, 27.8%, and 18.5% of the total volume change, respectively (p = 2.5 × 10^–4^, p = 0.018, and p = 0.018). The entirety of the expansion in matrix-like volume occurs in the uppermost five bins (the top 11% of the distribution). Striosome-like voxels did not differ significantly between ASD and TD at any point in the probability distribution. As noted above, there was no change in the volume of indeterminate voxels. This pattern of volume changes among the matrix-like, indeterminate, and striosome-like voxels is consistent with an isolated increase in the volume of the matrix compartment in ASD (Fig. 5C).

### The influence of experimental factors on matrix-like volume in ASD

Four experimental factors influenced the volume of the matrix-like compartment: diagnosis, hemisphere (L/R), striatal volume, and originating study. Sex and self-identified race had no impact on matrix-like volume (*p* = 0.14 for sex; p-values for race ranged from 0.11–0.39). The regression model including these four significant factors was moderately predictive of matrix-like volume (R^2^ = 0.32) but highly significant (F_(7, 844)_ = 57.8, *p* = 1.2 × 10^–67^). All regression coefficients for these variables were positive. The category of “autism”, as defined by high ADOS scores in the originating studies, correlated with increased matrix-like volume more strongly than the category of “autism spectrum disorders”, as defined by mid-range ADOS scores (t = 3.22 vs. t = 2.46; *p* = 0.0073 and p = 0.014, respectively), as distinguished by the diagnostic categories assigned by the originating studies. Note that we did not have sufficient ADOS data (measures of individual subjects) to compare matrix-like volume in those designated as “ASD” in the original studies with subjects whose ADOS scores fell in this mid-range. While matrix-like volume was significantly increased in ASD in both the left and right hemisphere, left sided striata were significantly more likely to be increased (left vs. right hemispheres, t = 2.00, *p* = 0.046). Of these four factors, total striatal volume had the most reliable, but quantitatively the smallest influence on matrix-like volume (t = 15.5, *p* = 5.6 × 10^–7^, coeff. = 0.373). Put simply, in most subjects larger striata included a larger number of matrix-like voxels, but this relationship had a smaller impact on inter-individual differences in matrix-like volume than the other three factors.

When assessing the interaction between diagnosis and striatal volume, matrix-like volume was positively correlated with striatal volume in all phenotypic categories (TD, ADOS-mid, and ADOS-high). However, regression coefficients suggested that increasing striatal volume had a slightly greater influence on matrix-like volume in ASD than in TD subjects (ADOS-high: 6.4% larger regression coefficient than TD, t = 16.2, *p* = 3.0 × 10^–4^); ADOS-mid: 3.9% larger regression coefficient than TD, t = 13.9, p = 4.0 × 10^–4^). Study-of-origin significantly impacted matrix-like volume for each study we included. However, the reasons for this impact were impossible to simplify to a single factor: the number of subjects included for a given study and the number of diffusion directions collected by that study had a linear relationship, and p-values for study-of-origin comparisons were therefore inversely correlated with the numbers of both subjects and sampled diffusion directions.

### Somatotopic influence on matrix-like volume in ASD

**Fig. 7:**
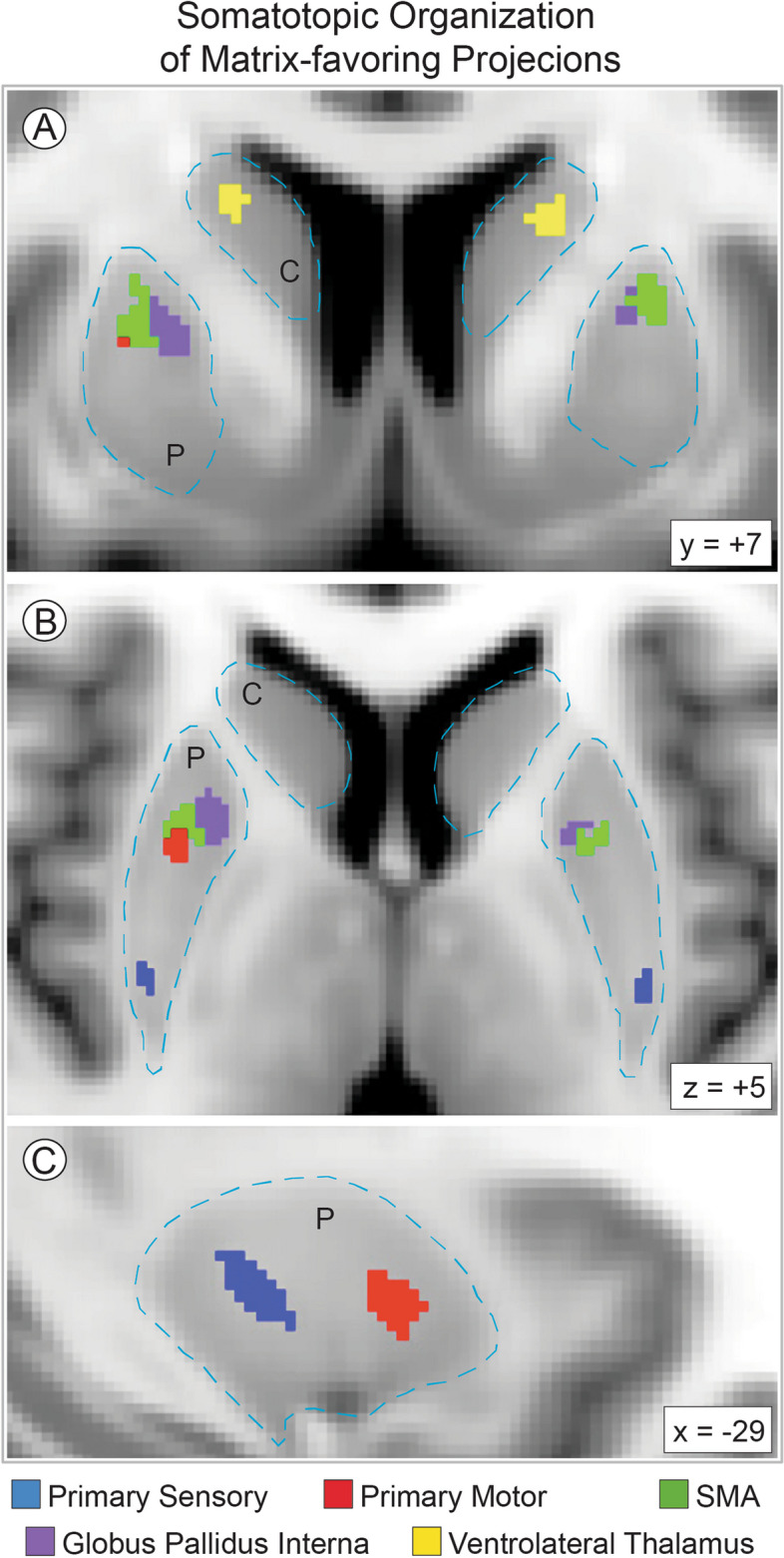
The Somatotopic Organization of Matrix-favoring Projections. Structural connectivity with the human striatum (outlined with light-blue dashes) is organized somatotopically. The five matrix-favoring bait regions utilized here project to all parts of the human striatum (caudate and putamen, indicated by C and P), but at particular zones (colored voxels), matrix-like connectivity is primarily driven by one bait region. Though the left and right hemispheres were parcellated independently, the location of their somatotopic zones was highly similar, as seen in axial (**A**), coronal (**B**), and left sagittal (**C**) planes. No cluster-forming algorithms were utilized. The five striosome-favoring bait regions also have distinct zones of influence, but only the matrix-favoring zones are shown here for clarity. While a somatotopic zone may abut zones specific for other matrix-favoring regions, there was no overlap in somatotopic zones. Somatotopic maps are overlaid on the MNI152_T1_1mm standard brain. Image follows radiographic convention. Coordinates follow MNI convention

**Fig. 8:**
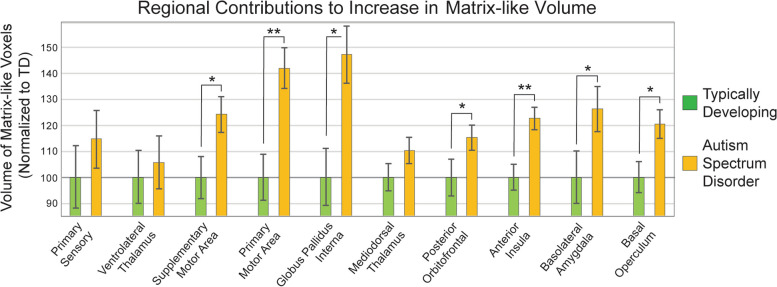
Regional Contributions to Matrix-like Volume. Mapping the somatotopic zones (seen in Fig. 7) where a given “bait” region had its greatest influence allowed us to quantify the contributions to matrix-like connectivity for each of the 10 bait regions we used to parcellate the striatum. The volume of matrix-like voxels (> 95th percentile) was expanded in ASD for each of the 10 bait regions (orange bars > green bars), though this volume exceeded our corrected significance threshold for only seven regions. Matrix-like volume was expanded throughout the striatum, in somatotopic zones for matrix-favoring regions and for striosome-favoring regions. For each somatotopic zone, volume was normalized to the typically developing (TD) cohort. **, *p* ≤ 3.0 × 10^–3^. *, *p* ≤ 3.0 × 10^–2^

While one can identify the striosome in every part of the striatum, they are concentrated in the rostral, medial, and ventral striatum [54, 56–58]. Layered atop these location gradients, projections from a particular extra-striate region to matrix or striosome are organized somatotopically [56, 58, 62, 63]. We mapped the somatotopic contribution of each of the 10 “bait” regions used to parcellate the striatum (test: hypothesis iv; Fig. 7). Notably, while we mapped left and right hemispheres independently, the somatotopic zones for a given bait region were highly similar between the hemispheres. We extracted the matrix-like volume (*P* ≥ 0.95) within each somatotopic zone to identify the bait regions with the largest contribution to increased matrix-like volume. In ASD, we found significantly increased matrix-like volume in 7 of 10 somatotopic zones (Table 1, Fig. 8), associated with the following bait regions: PMC, SMC, GPi, anterior insula, posterior orbitofrontal, basal operculum, and basolateral amygdala. Matrix-volume was increased in somatotopic zones specific for both matrix-favoring and striosome-favoring bait regions, and in all parts (rostral:caudal; dorsal:ventral) of both caudate and putamen. We wished to identify whether this bias toward matrix-like connectivity in ASD was limited to the striatum or was also present in the bait regions we utilized for striatal parcellation (test: hypothesis v). We performed quantitative probabilistic tractography between each bait region and the parcellated striatal compartments (equal-volume matrix-like and striosome-like voxels), and then compared ASD and TD on the number of voxels with high bias (*P* ≥ 0.95) toward matrix-like voxel targets. No bait region had a significant difference between ASD and TD in the bias toward matrix-like voxels, further suggesting that the expansion in matrix-like volume in ASD is driven by abnormalities within the striatum, not from abnormalities in the bait regions used to parcellate the striatum.

**Table 1:** Normalized matrix-like volume (± standard error of the mean) is expanded in autism spectrum disorder (ASD) relative to matched typically developing (TD) controls in somatotopic zones specific for each of the ten bait regions utilized for striatal parcellation. * indicates a significant increase in matrix-like volume

Somatopic Zone	Mean, Normalized Matrix Volume ± SEM	*p*-value, ASD vs. TD
Primary Sensory	115 ± 11%	0.21
VLc-VPLo Thal	106 ± 10%	0.35
Supplementary Motor	124 ± 6.9%	0.010*
Primary Motor	142 ± 7.8%	0.014*
Globus Pallidus Int	147 ± 11%	0.0062*
Mediodorsal Thal	110 ± 5.2%	0.11
Post. Orbitofrontal	115 ± 4.9%	0.0064*
Anterior Insula	123 ± 4.3%	0.010*
Basolateral Amyg	126 ± 8.8%	0.033*
Basal Operculum	120 ± 5.4%	0.0095*

## Discussion

ASD is a diverse collection of disorders with a large and growing number of genetic and injurious causes [11, 64, 65]. Identifying shared brain abnormalities is an essential step in understanding how these diverse etiologies can converge to the overlapping clinical phenotypes recognized as ASD. We found a selective elevation in the volume of matrix-like voxels that was widely distributed throughout the caudate and putamen. This increased volume was driven by increases in matrix-favoring bias within every bait region we assessed, with a high degree of left–right symmetry. Matrix-like volume was increased more in subjects with autism than in those with autism spectrum disorders, though this additional increase was associated with the category (autism) and not with the scale of autistic features (as estimated by calibrated severity scores). We found that subjects with ASD had modest decreases in streamline counts and slight decreases in FA, both a suggestion that the striatal compartments are structurally abnormal in ASD. However, neither of these abnormalities suggests a mechanism that would shift the matrix:striosome ratio. We conclude that the most likely source of these volumetric abnormalities is a developmental abnormality in ASD that leads to a widely distributed increase in matrix tissue. This conclusion matches the histologic findings of Kuo and Liu [35], though their assessment was limited to the rostral caudate and included only six pairs of human subjects. Expansion of the striatal matrix compartment, or more broadly, increasing the ratio of matrix:striosome function, is a neuroanatomic mechanism that could arise from inherited [66] or toxic [36] etiologies and would plausibly impact widespread functional brain networks [67]. Several important features of this hypothesis remain unanswered in the present study.

An increase in the matrix:striosome ratio could arise due to increases in matrix tissue or matrix-favoring connectivity, a decrease in striosome tissue or striosome-favoring connectivity, or a combination of these changes. Abnormalities in the striatal compartments may be restricted to particular zones, and due to the somatotopic organization of cortico-striate projections [59, 62, 68, 69], such a focused abnormality could selectively impact particular cortico-striato-thalamo-cortical loops. While each of these putative changes could increase the matrix:striosome ratio, and therefore contribute to ASD, it is plausible that the type and location of striatal tissue change would cause a distinct cluster of ASD features. The hypothesis that shifts toward matrix-facilitated striatal functions correlate with the clinical features of ASD is best tested in histology. Identifying the specific type of tissue changes associated with ASD is critical; since the striatum receives projections from nearly all cortical areas, it is uncertain why compartment disruption would specifically cause the unique features of ASD, rather than a more generalized developmental disability. Brain tissue samples in ASD may be limited in number and the parts of the striatum available for assessment. The large numbers of subjects available through neuroimaging studies may be useful to direct such histologic studies to particular regions of the striatum, or to tissue samples from particular subforms of ASD. Understanding the relative role of matrix and striosome in the development of ASD [35] will require histochemical assessments at a range of age points, sampling the striatum throughout its rostro-caudal extent, and in diverse causes of autism.

How might a shift toward matrix-like functions contribute to the diverse characteristics of autism? Striatal dopamine release increases firing in matrix MSNs but decreases firing in striosome MSNs [70], and in turn the compartments regulate nigral dopamine release and action selection through opposing mechanisms [29]. Given the key role of dopamine in learning and reward, individuals with ASD may find different behaviors salient or reinforcing. Since matrix and striosome are embedded in spatially-distinct cortico-striato-thalamo-cortical loops [71], an increase in matrix tissue may correlate with a selective augmentation of regions whose cortico-striate projections are biased toward matrix. Cortico-striate projections from primary sensory cortex, parietal operculum, posterior insula, and middle frontal gyrus strongly favor the matrix [58, 59, 62]. In each of these regions, unpleasant stimuli produce larger and quicker fMRI activation in subjects with ASD [72]. Similarly, primary motor cortex projections are highly biased toward matrix [58, 59, 68], and in ASD this region is functionally overconnected [73, 74]. Striatal functional networks evolve significantly throughout childhood [75, 76], suggesting that the timing of compartment-specific injuries, relative to the maturation of functional networks, may modulate the features that result. Cortico-striate projections in ASD may simply be less selective for either matrix or striosome. In the valproate rodent model of ASD [36], compartment boundaries are less distinct and stray matrix and striosome MSNs are localized in the opposing compartment. This loss of tissue segregation suggests a parallel loss of network segregation [67]. Such a striatum-wide histologic abnormality could underpin the motor, cognitive, and affective domains that are abnormal in ASD, but in different ratios in different autistic subtypes.

It is important to consider the limitations of probabilistic diffusion tractography, on which our findings are based. Tractography is blind to synapses, cannot distinguish afferent and efferent projections, and is susceptible to false positive and negative streamline estimations and inter-individual variance in streamline counts [77]. However, we used a combination of prior histologic studies in animals and hand segmentation of our anatomic masks to refine our tractography, increasing the anatomic plausibility of our findings: striatal parcellation has a test–retest error rate of 0.14% [58]; regions demonstrated to be matrix-favoring or striosome-favoring in animal tract-tracing studies show the same biases in living humans [58, 69, 71]; these effects are specific for our precisely-selected compartment-like voxels, as shifting their position by just 2–3 mm completely negates these regional biases [71]. Another limitation of our parcellation method is the mismatch between our diffusion voxel size (2 mm isotropic) and the upper limit of striosome diameter (approximately 1.25 mm [54, 55]). Even perfectly-centered striosome branches will be averaged with some volume of surrounding matrix, diluting between-compartment differences. While MRI resolution is a limitation, our matrix-like and striosome-like voxels replicate the patterns of spatial distribution, relative abundance, and region-specific connectivity of matrix and striosome tissue demonstrated in animal and human histology [58, 69, 71]. Despite the strength of these findings, readers should recall that our method identified voxels that share features with the striatal compartments – hence are matrix-like and striosome-like – but this inferential process did not directly identify matrix or striosomes.

While our MRI-based method has clear limitations, identifying matrix and striosome directly requires *post mortem* tissue in humans, precluding compartment-specific investigations of function. Distinguishing compartment-like voxels in vivo offers several advantages in the study of ASD and suggests future directions for inquiry. Comparing the parcellated striata of groups of individuals whose ASD manifests in different ways – such as those with primarily repetitive movements vs. those with selective communication impairment – may reveal differences in where or how the matrix:striosome ratio is different from those without ASD. Identifying the striatal locations where matrix:striosome ratios are abnormal, and the tissue-level change that led to abnormal compartment ratios (increased matrix vs. decreased striosome), may explain some of the variance in the autism spectrum. Parcellated striatal voxels can serve as the seeds for functional connectivity [67]. For example, assessing the emotional content of faces, extracting affective cues from language, and weighing risk-reward scenarios are behavioral tasks that may activate striosome-like voxels more than matrix-like voxels. In contrast, repetitive movement tasks and sensory stimulation tasks may activate matrix-like voxels more than striosome-like voxels. Longitudinal studies of patients and younger siblings are necessary to evaluate the influence of the striatal compartments on abnormal developmental trajectories in ASD [78]. Such comparisons may reveal whether abnormal compartment ratios precede or parallel deviations from typical development. Nearly all parts of the human diencephalon and telencephalon have afferent or efferent connections with the striatum, and for most regions connectivity is biased toward one compartment [58]. Our scant knowledge of matrix- and striosome-specific biology is a major limitation in understanding how the striatal compartments influence the characteristics of ASD.

## Supplementary Information

Supplementary Material 1.

## Data Availability

We accessed MRI and clinical testing data through the National Institutes of Mental Health Data Archive (NDA) portal. This cohort can be accessed through a study specific identifier (10.15154/z7aa-pz74). Linux code for generating and processing tractography is available at github.com/jeff-waugh/Striatal-Connectivity-based-Parcellation.
