# Supplementary material for: The striatal matrix compartment is expanded in autism spectrum disorder
Source: J Neurodev Disord. 2025 Feb 15;17:8. doi: 10.1186/s11689-025-09596-7 (PMC11829417; doi:10.1186/s11689-025-09596-7)
Supplement: Supplementary file 1 — Supplementary Material 1. [file 11689_2025_9596_MOESM1_ESM.docx]

MRI Acquisition
 Participants in all cohorts were imaged using 3T high-resolution whole brain diffusion tensor imaging (DTI) sequences. While most scans were collected at 2 mm isotropic resolution, one study collected DTI at 2x2x2.5 mm [43]. We resampled those images to 2 mm isotropic prior to processing. Though we selected studies whose imaging parameters were similar, the details of these dMRI sequences differed between studies, underscoring the importance of maximizing intra-study matching for ASD:TD pairs. We refer readers to the original studies for details of the dMRI sequences utilized [41-44].

DTI preprocessing
 We processed DTI images with the FSL FMRIB Diffusion Toolbox (Release 6.0, 2018, http://fsl.fmrib.ox.ac.uk/fsl/fslwiki) using standard parameters. Preprocessing for each subject included radiological orientation, removal of non-brain tissue (*bet2*), correction for head motion and eddy current distortions (*eddy_openmp*). We estimated diffusion tensors at each voxel (*dtifit*) and calculated diffusion probability estimates at each voxel using a Markov Chain Monte Carlo procedure (*bedpostx*). We generated registration matrices from MNI space into native diffusion space for each subject using the FSL linear and non-linear registration tools, *flirt* and *fnirt*. We first registered each subject’s native space FA image to the FMRIB58_FA_1mm template (MNI space), then inverted this registration matrix using *invwarp* to generate an MNI-to-Native registration matrix. We utilized these registration matrices to generate seed and exclusion masks for probabilistic tractography in native space, and the inverse of these matrices to register the products of tractography into MNI space for voxelwise comparisons and generation of group averages.

Anatomical Segmentation of Regions of Interest
 The regions utilized in this manuscript and the methods for their manual segmentation were described in our prior publication [45]. Briefly, we segmented regions of interest (ROIs) on the MNI152_T1_1mm template brain using the atlases of Talairach and Tournoux [46] and Mai et al. [47] to produce accurate and reliable segmentations. We then compared each segmentation with the FMRIB58_FA_1mm template brain to ensure a cross-modality match. We transformed each standard space ROI into each subject’s native space to act as seed, target, or exclusion masks for probabilistic tractography. Our striatal segmentation combined the caudate and putamen, excluding the nucleus accumbens and the caudal third of the tail of the caudate, beyond the posterior extent of the putamen, as our prior experience suggested that the small cross-sectional area of the posterior-most caudate tail in the axial plane is difficult for successful automated registration using 2 mm isotropic images. A midline exclusion mask, which blocked all interhemispheric projections (including mis-tracked interhemispheric streamlines), was centered on the sagittal plane and allowed us to focus on projections within the ipsilateral hemisphere.
